# The Hunt for Natural Skin Whitening Agents

**DOI:** 10.3390/ijms10125326

**Published:** 2009-12-10

**Authors:** Nico Smit, Jana Vicanova, Stan Pavel

**Affiliations:** 1 Department of Clinical Chemistry, room L02-56, Leiden University Medical Center, P.O. Box 9600, 2300 RC Leiden, The Netherlands; 2 DermData, Prague, Czech Republic; E-Mail: JV@derm-data.com (J.V.); 3 Department of Dermatology, Leiden University Medical Center, P.O. Box 9600, 2300 RC Leiden, The Netherlands; E-Mail: S.Pavel@lumc.nl (S.P.)

**Keywords:** whitening, tyrosinase inhibitors, natural agents, cosmetics

## Abstract

Skin whitening products are commercially available for cosmetic purposes in order to obtain a lighter skin appearance. They are also utilized for clinical treatment of pigmentary disorders such as melasma or postinflammatory hyperpigmentation. Whitening agents act at various levels of melanin production in the skin. Many of them are known as competitive inhibitors of tyrosinase, the key enzyme in melanogenesis. Others inhibit the maturation of this enzyme or the transport of pigment granules (melanosomes) from melanocytes to surrounding keratinocytes. In this review we present an overview of (natural) whitening products that may decrease skin pigmentation by their interference with the pigmentary processes.

## 1. Introduction

In the skin, melanocytes are situated on the basal layer which separates dermis and epidermis. One melanocyte is surrounded by approximately 36 keratinocytes. Together, they form the so-called epidermal melanin unit. The melanin produced and stored inside the melanocyte in the melanosomal compartment is transported via dendrites to the overlaying keratinocytes. The melanin pigment is a polymer produced inside the melanosomes and synthesised from the amino acid l-tyrosine that is converted by the enzyme tyrosinase to dopaquinone [1]. This reaction continues spontaneously via dopachrome to the monomeric indolic precursors (5,6-dihydroxyindole and 5,6-dihydroxyindole 2-carboxylic acid) of the black-brown pigment eumelanin. However, some other enzymes, like the tyrosinase related proteins (TRP-1 and dopachrome tautomerase (TRP-2) may also play an important role in melanogenesis *in vivo*. Upon reaction with cysteine, dopaquinone forms 2- or 5-*S*-cysteinyldopa that generates the benzothiazine precursors of the red/yellow pheomelanin polymer. In general, a mixed type of pheo- and eumelanin polymer is produced and deposited onto the melanosomal matrix proteins. Considering the many colour variations that can be seen in the skin and hair, one may expect that the composition of the mixed melanins is regulated in many different ways. However, altered production of cutaneous melanin may cause considerable problems of esthetic nature, especially in hyperpigmentary conditions, like melasma, postinflammatory hyperpigmentation, freckles or lentigines. But also depigmenting conditions, like vitiligo, have high impact on the quality of life of the patients.

In the Western culture it is still considered desirable to obtain a (bronze) tan. Despite warnings about the consequences of excessive sun or UV exposure, the artificial tanning business has expanded strongly in the last decades. In the Eastern world, however, a centuries long tradition exists whereby a light complexion is regarded as equivalent to youth and beauty. Development of preparations for bleaching hyperpigmented lesions or to safely achieve overall whitening is one of the challenges for cosmetic industry. In recent years, the interest in skin whitening has grown tremendously.

## 2. Targeting Tyrosinase as the Key Enzyme of Melanogenesis

One of the most obvious cellular targets for depigmenting agents is the enzyme tyrosinase. The scientific literature on tyrosinase inhibition shows that a large majority of the work has been conducted since 2000 and has mostly been devoted to the search for new depigmenting agents. Notably, many of these studies deal with tyrosinase inhibitors from natural sources and are mostly of Asian origin (see Tables 1 and 2). However, early pioneering work in the field has been performed much earlier using 4-hydroxyanisole. This compound could serve as an alternative substrate for tyrosinase causing depigmentation both *in vivo* and *in vitro* [2,3]. Since this and various other substituted phenolic compound can generate potentially toxic quinone products they were used in various studies aimed at the induction of toxicity mediated by tyrosinase in melanoma cells [4,5].

Considerable interest in tyrosinase inhibitors exists also in the food industry because the activity of this enzyme is responsible for the browning of fruit and vegetables. Cysteine or ascorbic acid can be used to prevent the enzymatic browning of fruit and vegetables by binding the *o*-dopaquinone intermediates. More recently also 4-hexylresorcinol has been utilized for this purpose [6–9]. Since safety considerations are very strict in food industry, the search for new, natural tyrosinase inhibitors without negative side effects is of utmost importance in this field of research.

Work on synthetic and natural tyrosinase inhibitors has been recently reviewed in several papers [7,9,10]. The tyrosinase inhibitors can be classified as competitive, uncompetitive, mixed type and non-competitive inhibitors [10]. The nature of tyrosinase inhibition can be disclosed by measuring enzyme inhibition kinetics using Lineweaver-Burk plots with varying concentrations of l-DOPA as the substrate. This can be seen on example of polyphenol extracts from acerola (West Indian cherry) or a chalcone derivative isolated from *Morus nigra* (black mulberry) which has been described in recent work of Hanamura *et al*. and Zhang *et al*. [11,12]. Knowledge of the type of inhibition may be important in order to achieve better skin lightening effects since combined treatments may result in synergistic effects. This has been shown in case of the competitive tyrosinase inhibitor, arbutin and the noncompetitive inhibitor, aloesin [9,13].

A 2009 paper by Chang states that a large majority of tyrosinase inhibitors show reversible inhibition [10]. In irreversible inhibition, covalent binding with the enzyme may cause its inactivation by altering the active site of the enzyme and/or by conformational changes to the protein molecule. Irreversible inhibition may also occur via the so-called suicide inhibition mechanism as described in the model by Land *et al*. [14]. Also, two 8-hydroxy isoflavones isolated from soygerm koji showed suicide inhibition of tyrosinase and have been tested with promising results in an *in vivo* assay with 60 volunteers [10]. In Table 1 we summarize the large number of studies using tyrosinase inhibitors from natural sources that have appeared, mostly in the last decade. In many of the investigations, the active ingredients from extracts of various species have been isolated and identified. In case the mode of tyrosinase inhibition was established, a comparison with IC_50_ values of well known inhibitors such as kojic acid and arbutin was often made. In some of the studies specific side groups (with substitutions to C4, C5 or C8 position) of recorcinols isolated from the breadfruit (*Artocarpus incisus*) or from a ‘bitter root’ (*Sophora flavescens*) proved of great importance to their inhibitory potential [15,16]. In some cases modifications to the natural compounds were made, e.g., the deglycosylation of stilbene compounds by cellulase treatment of the *Veratrum patulum* extract resulted in improved tyrosinase inhibition [17]. Thus, exact knowledge on enzyme inhibition mechanisms is helpful for designing new whitening products based on targeting the key enzyme of melanogenesis, tyrosinase. Although tyrosinase plays a major role in melanin synthesis, one should realize that the regulation of skin pigmentation exists at various levels and therefore, different modes of interference are possible. There are indications that combined approaches could be more successful than targeting tyrosinase only.

TI; tyrosinase inhibition, (c) competitive mode (nc) non competitive mode of inhibition. SB; Streptomyces bikiniensis [47]. MMS; molecular modeling studies on TI. SAR; structure activity relationship. PI; pigment inhibition.

Tyrosinase inhibition among different studies is difficult to compare for several reasons (see also Chang [10]) because of different sources of tyrosinase used (see Parvez, [9]) and IC_50_ values that are found using either tyrosinase or l-DOPA as the substrate. In the table comparison to kojic acid (KA) for some of the component (number) is indicated as < or > or compounds are compared among each other (1 > 2).

Extraction procedures for isolation and identification are highly important for good yield of the active ingredients. Many of the papers in Table 1 describe different extraction procedures. An overview of TI from natural and synthetic sources has been presented earlier in the review by Kim and Ujama [7].

## 3. Different Modes of Reducing Melanin Production in Melanocytes and Skin

As proposed by Briganti *et al*. all depigmenting agents may be divided on the basis of interference in melanin synthesis, transport and removal by skin turnover [48]. In Table 2, we sum up a large number of studies that describe new whitening agents from natural sources with some extra information on their mode of action besides the inhibition of tyrosinase. Next to tyrosinase inhibition (TI) the extracts or their isolated active components were demonstrated to exhibit pigment inhibition (PI). For this purpose, some studies make use of the pigment-producing *S. bikiniensis* (SB) system [37,49] or transformed *E.coli* [32]. In most cases, however B16 melanoma cells are used for demonstrating PI. In addition, PI is demonstrated in the mouse melan-a or mel-ab melanocyte cultures or in normal human melanocytes (nHEM). Obviously, the use of the nHEM may better simulate the *in vivo* situation. On the other hand, the melanocytes are more difficult to maintain in culture. These cells, also show variations in melanin content from donor to donor and from one passage to the other [50]. Cocultures of melanocytes and keratinocytes from mouse [51,52] or human skin [53] more closely mimic the *in vivo* situation and, eventually, a skin equivalent model (SEM) may be the preferred *in vitro* system for testing skin whitening agents [54]. In this respect, recently commercially available SEM have already been applied for skin whitening studies [55]. Next to this, the brownish guinea pig (GP) model is used in several studies (Table 2) where the pigmentation is induced by either UV or α-MSH. In case of *in vivo* studies, prevention of the induction of pigment by the whitening agents could be demonstrated using a Minolta chromameter or by histochemical investigations showing a decrease in DOPA positive cells [56,57]. Another animal model used for whitening studies is the zebrafish that also proved useful for demonstrating the *in vivo* toxicity of the whitening agents [58,59]. So far, only limited numbers of clinical trials (CT) with skin whitening agents or formulations have been performed [10,60].

Preventing the maturation or intracellular trafficking of tyrosinase is an alternative way to reduce the effect of the enzyme on pigmentation [61–63]. Various natural extracts can also influence tyrosinase mRNA at the transcription level; also mRNA of the other tyrosinase-related proteins or microphtalmia transcription factor (MITF) can be affected (see refs. [59,64,65] and others in Table 2). From the work of Sharlow *et al*. [66] and Seiberg [67] we learned that the protease activated receptor 2 (PAR-2) is important for melanosomal transfer from melanocytes to keratinocytes and that this transfer can be used as a target for skin lightening [68]. The vitamin B3 derivative niacinamide is one of the agents used for inhibiting melanosomal transfer [53]. Melanocytes express high levels of sAPP, the soluble *N*-terminal ectodomain of the β-amyloid precursor protein [69]. sAPP may play a role in the release of melanin particles via dendritic tips. Blocking the sAPP signalling could thus be another way to influence melanosome transport.

Mammone *et al*. [70] (Estee Lauder) proposed that melanin can be degraded enzymatically in keratinocytes and application of melanin degrading enzymes could be used to prevent UVB induced pigmentation in human skin.

Reduction of ROS levels in melanocytes may prevent activation of melanogenesis. In various studies, extracts from plants or fruit or other species were tested for their antioxidant capacity by using the 1,1-diphenyl-2-picrylhydrazyl (DPPH) radical-scavenging assay or the oxygen radical absorbance capacity (ORAC) (e.g., Rangkadilok *et al*. [71]). Fujiwara and colleagues [72] showed that daily oral administration of vitamin C (ascorbic acid) and vitamin E and cysteine to brownish guinea pigs reduced UVB-induced pigmentation. Ascorbic acid is considered a skin whitening agent and more stable derivatives such as ascorbyl glucoside and ascorbyl palmitate are already being used in different skin whitening formulations [73]. As known from many cases of post-inflammatory hyperpigmentation, melanogenesis can be stimulated by some inflammatory mediators. Inhibition of the production of inflammatory mediators (Il1α and TNF-α) was reported for sea grape extracts [74]. Via this indirect way stimulation of melanogenesis in the pigment cells could be prevented [48].

## 4. Induction of Pigmentation

For the development of effective skin whitening, we also need to understand processes that regulate the induction of pigmentation. Constitutive pigmentation is reflected by the phenotypes of the different skin types with varying pigmentation based on their genetic diversity. The facultative pigmentation acquired on top of the constitutive level can be obtained via different stimuli of which ultraviolet radiation (UVR) is well known as provoking the “tanning response”. An overview of the signalling pathways and intrinsic and extrinsic factors (inclusive UV) that influence melanocyte proliferation or metabolism can be found in the paper by Brenner and Hearing [109]. In brief, the UV response increases the microphtalmia-associated transcription factor (MITF) that is on its turn regulated by another transcription factor SOX9 [110]. MI is the main switch for induction of the melanogenic proteins responsible for the final increase of the melanin content in skin after UV exposure. Various pathways can be induced by the signalling through basic fibroblast growth factor (bFGF), hepatocyte growth factor (HGF), stem cell factor (SCF), endothelin-1 (ET-1), adrenocorticotropic hormone and α-melanocyte stimulating hormone (ACTH and α-MSH) via their respective receptors present on melanocytes and thus stimulating their pigment production. These signalling pathways could also serve as a means of specific targeting the melanogenic pathway. In this way, the presence of melanocortin-1 receptor (MC1R) on (B16) melanoma cells has been often used for induction of pigmentation and for testing the depigmenting effects of natural skin lighteners (see examples in Table 2).

Several authors focus on factors that were not directly involved in melanin synthesis but could affect proteins indirectly connected with skin pigmentation. For instance, the endothelin-1 induction of pigmentation in melanocytes could be prevented by 3’antisense S-oligo for tyrosinase that also reduced UV induced pigmentation [111].

The Wnt/β-catenin pathway is known to play an important role in developmental processes [112]. Binding of Wnt proteins to their receptors (the *frizzled* family of transmembrane proteins)can be inhibited by Dikkopf 1 (DKK1), a factor secreted by fibroblasts which can suppress growth of melanocytes and strongly inhibit melanin production [109,113]. Thus, some of the natural whitening agents presented in Table 1 or 2 are not direct inhibitors of tyrosinase but downregulate expression of melanogenic proteins and in this way they may interfere with the complex regulation of melanocyte signalling cascades. Stem cell factor is a cytokine that binds to the c-kit receptor (CD117) and the activation of c-Kit leads to the activation of multiple signaling cascades, including the RAS/ERK, PI3-Kinase, Src kinase, and JAK/STAT pathways [114]. Na and coworkers [115] have used the signalling via SCF/c-kit for the evaluation of new whitening agents by high throughput screening with approximately 10.000 synthetic compounds. They found that phenyl-imidazole sulfonamide derivatives prevented stem cell factor induced c-kit phosphorylation in (501mel) human melanoma cells and also the UV induced pigmentation on brownish guinea pigs. Furthermore, the SCF/c-kit pathway was used to induce pigmentation in case of vitiligo. Geniposide (from the fruit of *Gardenia jasminoides Ellis*) is used in traditional Chinese medicine for treatment of generalized vitiligo. This compound was shown to increase pigmentation via SCF/c-kit in normal human melanocytes where melanogenesis was suppressed by norepinephrine [116]. In the case of SOX9 and MITF, signalling is mediated via cAMP and PKA [110], but also the stimulation of PKC (via diacylglycerol and calcium) may results in activation of tyrosinase. Inhibition of PKC by the specific PKC inhibitor bisindolylmaleimide (bis) resulted in a reduced tanning response in pigmented guinea pigs and in a marked lightening of freshly depilated hairs in mice [117].

Furthermore, Yaar *et al*. [118] proposed bone morphogenetic proteins (BMPs) to be involved in modulating melanogenesis since melanocytes express the BMP receptors and produce BMP-4, that is able to decrease melanin synthesis in human melanocytes in culture. Another mechanism of pigment regulation is suggested for the peroxisome proliferator- activated receptor (PPAR) since binding of octadecenedioic acid to this PPAR leads to reduced melanogenesis and tyrosinase expression. The same was found for a known pharmaceutical PPAR agonist rosiglitazone [119].

Another pathway indicated in several papers by Kim *et al*. is the signalling via extracellular signal-regulated kinases (ERK). This pathway can be triggered by different stimuli, like growth factors (bFGF and HGF) and cytokines (SCF), as indicated above. The authors first reported that c2-ceramide inhibits melanogenesis by activation of ERK and they showed that inhibition of ERK (and AKT/PKB) caused an increase in pigmentation in human melanocytes [120]. As a follow-up they described a new 2-imino-1,3-thiazoline derivative that decreased melanin production in B16 melanoma cells via induction of ERK [121]. More recently they showed that terrein, which acted on ERK and downregulated MITF, in combination with a new tyrosinase inhibitor, KI-063, caused additive effects on depigmentation in the Mel-ab melanocytes [122]. They also described a new imidazole derivative AVS-1357 that reduced pigmentation by activation of ERK and the downregulation of MITF and tyrosinase [123]. Similar results were achieved with haginin A that inhibited tyrosinase and also activated ERK and thus downregulated MITF and tyrosinase and TRP-1. Haginin A effectively reduced pigmentation in the brownish guinea pig and the zebrafish model systems [59].

The effects of ceramide on pigmentation is of interest as well since it has been reported that glycosylation of lipids could be of importance for proper sorting of the melanogenic proteins to the melanosomes [124]. A glycosphingolipid-deficient melanoma culture was not pigmented and by transfection with ceramide glucosyltransferase, pigmentation could be restored [124]. We found that reducing the levels of glucosylceramide may affect pigment production in normal human melanocytes. In this respect it is interesting to note that 1-deoxynojirimycin (DNJ) is a glycosidase inhibitor and one of the main components in mulberry leaves (from *Morus alba*) [125] and personal communication Aerts JMG, Academic Medical Center, University of Amsterdam). As shown in Tables 1 and 2 the compounds isolated from *Morus alba* (oxyresveratrol, mulberroside F and betulinic acid) inhibited tyrosinase [41,82,83] but the effect of DNJ on lipid glycosylation could inhibit melanin synthesis as well (N. Smit, manuscript in preparation).

## 5. Cosmetic Use of (natural) Agents for Skin Whitening

In cosmetic formulations hydroquinone (HQ) has been widely used as an effective whitening agent but it has been banned recently because of serious safety concerns: its use has been connected with mutagenicity and the increased incidence of ochronosis in African countries. Other compounds often used are kojic acid, arbutin and azelaic acid (see top of Table 2). Arbutin is a glycosylated form of HQ that is present in bearberry extracts but it can also be synthesized from HQ by glucosidation. A new derivative, deoxyarbutin was prepared by removal of all hydroxyl groups from the glucose side chain of arbutin and showed much lower cytotoxicity than arbutin [126,127]. In the large variety of whitening products, nowadays commercially available the use of different natural whitening agents is noticeable. Although the information on the exact formulations for all the whitening products is not easily accessible on the internet, we made an attempt to summarize the active whitening ingredients for some of them (Table 3). The utilization of kojic acid and arbutin is still common because these agents have repeatedly been demonstrated to be effective whitening agents. The use of bearberry extracts (a natural source of β-arbutin) may strengthen the effect of α-arbutin in Meladerm and Lucederm preparations. Among the natural extracts, mulberry and licorice are popular components added to the skin whiteners. The isolation of their active components and their ffect on tyrosinase inhibition (TI) and pigment reduction (PI) has been described (see Tables 1 and 2). Also lemon extract is used in the preparations like Skin Bright, Lucederm and Meladerm as a potent skin bleaching ingredient. However, it can only be used at low concentrations because it easily causes skin irritation. In Tables 1 and 2 several studies are included describing *Sophora* species from which several active compounds have been isolated that act as potent inhibitors of tyrosinase and pigment production. Also in the product Synerlight from LiBiol an extract from *Sophora* species is present. In this case it is combined with Kiwi fruit (*Actinidia Chinensis*) which contains flavonoids (e.g., quercetin) that may be responsible for tyrosinase inhibition [10]. Niacinamide, which besides inhibition of tyrosinase, interferes in melanosome transfer to keratinocytes is used in the formulations of Meladerm and Lucederm. The Revitol product Skin Brightener contains Lumiskin with some patented ingredient, diacetyl boldine, that influences tyrosinase at the expression level. The Mandresy extract of Bayer contains two compounds luteolin and verbascoside that do not only inhibit tyrosinase and pigment production but also influence the interaction between keratinocytes and melanocytes by reducing formation of dendrites. Some of the products (Meladerm and Tosseki whitening cream) contain a mixture of many extracts with the obvious tyrosinase inhibitors (Mulberry, Licorice, Sophora and Peonia) but also other extracts that may act as antioxidant or anti-inflammatory. One of the components of the Meladerm preparation is TegoCosmo which contains a guanidine compound that acts on tyrosinase activity. Another component is Gigawhite that contains various plant extracts from the Alps and that has been tested on 10 subjects of Asian origin. Its bleaching effects may partly be attributed to tyrosinase inhibition. The question arises whether the increasing amounts of potentially active whitening ingredients will cause additive effects or will reduce the effects of the most potent ingredients (by competitive inhibition).

Some companies still use single synthetic compounds. For instance Lipotec uses dimetylmethoxy chromanyl palmitate in its product Chromabright. This exhibited lightening activity in a group of 20 Asian volunteers after 30 and 60 days. Sederma company makes use of a new mechanism of action targeting the peroxisome proliferator- activated receptor (PPAR). Their active ingredient named O.D.A. White is able to reduce tyrosinase mRNA expression [119].

Thus, approaches for skin whitening have broadened widely in the recent years. The utilization of single agents inhibiting tyrosinase is in many cases extended to the use of complex mixtures that target different mechanism like tyrosinase expression, transfer of melanosomes, antioxidant and anti-inflammatory effects.

## Figures and Tables

**Table 1.** Compounds selected as tyrosinase inhibitors by extraction from natural sources and the (possible) isolation and characterization of the active ingredients.

**Source**	**Compounds (type)**	Mode of action tested*		**Refs.**
		**TI**	**comments**	

Chouji and Yakuchi extracts, crude drugs	eugenol, yakuchinone A, ferulic acid, curcumin and yakuchinone B	TI (c)		[18]

*Anacardium occidentale*, cashew fruit	6-[8(*Z*),11(*Z*),14-pentadecatrienyl]-salicylic acid and 5-[8(*Z*),11(*Z*),14-pentadecatrienyl]resorcinol	TI (c)		[19]

Bolivian medicinal plants, *Buddleia coriacea*, *Gnaphalium cheiranthifolium*, and *Scheelea princeps*.	phenolic	TI		[20]

*Artocarpus gomezianus*.	among eight other compounds norartocarpetin (5) and resveratrol (8) were isolated	5,8 were most potent TI		[21]

*Artocarpus incisus*	flavonoids, stilbenes and related 4-substituted resorcinols	TI	4-substituted recorcinol increases TI (c)	[15]

*Stryphnodendron barbatimao*, *Portulaca pilosa*, *Cariniana brasiliensis*, *Entada africana* and *Prosopis africana*. Five plants out of 67 tropical plants	unknown	strong TI	TI comparable to *Morus alba* as positive control	[22]

*Pulsatilla cernua*	3,4-dihydroxycinnamic acid (1) 4-hydroxy-3-methoxycinnamic acid (2)	2 > other TI > 1 1,2 (nc)		[23]

galls of *Rhus javanica*	Tannic acid	TI	-	[24]

*Sophora flavescens*	prenylated flavonoids; kuraridin, kurarinone and norkurarinol	strong TI > KA	C8 and C5 substitutionis essential for TI	[16]

*Sophora flavescens*	sophoraflavanone G, kuraridin, and kurarinone	TI > KA		[25]

*Veratrum patulum*	hydroxystilbene compounds; resveratrol, oxyresveratrol, and their analogs	potent TI	cellulase treatment improved TI	[17]

*Phellinus linteus*	cerebroside B (1), protocate-chualdehyde (2), 5-hydroxymethyl-2-furaldehyde (HMF) (3), succinic acid (4), fumaric acid (5)	2,3 TI 2 > 3 2 (c) 3 (nc)		[26]

*Ecklonia stolonifera*. edible brown alga out of 17 seaweed extracts	phloroglucinol derivatives [phloroglucinol (1), eckstolonol (2), eckol (3), phlorofucofuroeckol A (4), and dieckol (5)].	1,2 TI (c) 3–5 (nc)	TI similar to arbutin	[27]

39 seashore plant species, Japan: *Hibiscus tiliaceus*, *Carex pumila*, and *Garcinia subelliptica*	GS contained 2 biflavonoids; 2*R*,3*S*-5, 7,4′,5″,7″,3‴,4‴-heptahydroxy-flavanone[3-8″] flavone (1) and 5,7,4′,5″,7″,3‴,4‴-heptahydroxy[3–8″] biflavanone (2)	both strong TI 1 > KA		[28]

*Glycyrrhiza uralensis Glycyrrhiza inflate* Licorice	liquiritin(1), licuraside (2), isoliquiritin(3), liquiritigenin(4) and licochalcone A (5)	2,3 and 5 potent TI (c)		[29]

*Trifolium balansae*	three steroids, stigmast-5-ene-3 beta,26-diol (2), stigmast-5-ene-3-ol (3) and campesterol (4)	2,3 and 4 potent TI 2 > 3,4		[30]

*Amberboa ramosa* Jafri	cycloartane type triterpenoids; eight compounds identified. 3β,21,22,23-tetrahydroxycycloart-24 (31),25(26)-diene (cmpd. 7)	7 most potent TI > KA	SAR studies	[31]

*Sake lees*	triacylglycerols; triolein (1) and trilinolein (2)	TI 1,2 (nc) TI 2 > 1	PI in *E coli* (2)	[32]

*Garcinia kola*	screening 21 families of medicinal plants from West and Central Africa. 5 extracts selected with G. kola showing good TI; five biflavanones identified	TI > 60% IC50 > KA		[33]

*Marrubium velutinum* and *Marrubium cylleneum*	Screening of 45 metabolites. Flavonoids (1), phenylethanoid glycosides (2), phenolic acids (3)	1,2 moderate TI, 3 < 2		[34]

Lichen species; Graphidaceae family(1) *Usnea ghattensis* (2), *Heterodermia podocarpa*, *Arthothelium awasthii* (3) and *Parmotrema tinctorum*	unknown	TI (1) 30–78%	antioxidant, antimicrobial, antityrosinase IC50 (2,3) similar or less than other TIs	[35,36]

*Sophora flavescens*	sophoraflavanone G (1), kurarinone (2) and kurarinol (3)	strong TI 1,2 (nc) 3 (c)	1,2 antibacterial 3 PI in SB MMS on 3	[37]

50 crude drugs *Glycyrrhiza glabra*, *Morus alba*, *Syzygium aromaticum*, *Citrus aurantifolia*, *Cypreae moneta*, *Punica granatum* and *Citrus aurantium*		yes All < KA		[38]

*Ganoderma lucidum*		yes		[39]

*Arbutus andrachne*	Arbutin, hydroquinone, β-sitosterol and ursolic acid present in extracts	yes		[40]

*Morus alba* L. and *Morus rotundiloba* Koidz Mulberry	betulinic acid (present)	yes	anti inflammatory	[41]

*Guioa villosa*	sesquiterpene diglycosides; crenulatosides E, F and G (1 – 3) betulin (14), lupeol (15) and soyacerebroside I (16)	no strong TI		[42]

*Broussonetia kazinoki.*	1,3-diphenylpropanes: kazinol C (1), D (2), F (3), broussonin C (4), kazinol S (5) and kazinol T (6)	1,3–5 (c) 4; max TI	- -	[43]

*Artocarpus heterophyllus*	15 compounds. norartocarpetin (4) and artocarpesin (6)	yes 5 cmpds > KA	- -	[44,45]

*Paeonia suffruticosa*	kaempferol (I), quercetin (II), mudanpioside B (III), benzoyl-oxypaeoniflorin (IV), mudanpioside H (V), and pentagalloyl-β-d-glucose (VI)	yes I to V (c) VI (nc)	- -	[46]

**Table 2.** New whitening agents from natural sources and their mode of action as tyrosinase inhibitor (TI), inhibitor of pigment synthesis (PI) or by other mechanisms. Azelaic acid, Kojic acid, Arbutin and Aloesin are often used as positive skin whitening agents.

**Source**	**Compounds (type)**	Mode of action tested(*)(**)			**Refs.**
		**TI**	**PI**	**other**	
*Pityrosporum ovale*	Azelaic acid; C9-dicarboxylic acid	yes	yes	-	[75]
*Aspergillus niger* and *Aspergillus penicillium*	Kojic acid; 5-hydroxy-2-(hydroxymethyl)-γ-pyrone	Yes (c,m)	-	-	[76,77]
*Arctostaphylos uva*-ursi bearberry	Arbutin; hydroquinone glucoside β-d-gluconopyranoside	yes (c,m,nc)	-	-	[13,78] [79]
*Aloe vera*	Aloesin; C-glycosylated chromone	yes (nc)	-	-	[9,13,80]
*Artocarpus incisus* (best of) 23 heart wood species from Papua New Guinea.	(+)-dihydromorin, chlorophorin, (+)-norartocarpanone, 4-prenyl-oxyresveratrol, artocarbene, artocarpesin and isoarto-carpesin	yes ≈ KA	yes (B16 and GP)	-	[81]
*Morus alba Rheum undulatum*	1. Oxyresveratrol 2. Hydroxystilbene	yes > KA (nc) yes	-	1. no effect on expression or synthesis	[82]
*Morus alba*	Mulberroside F (moracin M-6, 3′-di-O-beta-d-glucopyranoside	yes	yes (melan-a)	mild anti-oxidant SO scavenger <KA	[83]
Citrus fruit peel	3′,4′,5,6,7,8-hexamethoxy-flavone (nobiletin)	yes > KA		antimutagenic	[84]
*Ramulus mori* (young twigs of *Morus alba*)	2,3′,4,5′-tetrahydroxy-stilbene (2-oxyresveratrol)	yes (c)	yes (GP + UV)	no effect on expression or synthesis non-toxic	[85]
*Glycyrrhiza glabra* Licorice extract	glabrene and 2′,4′,4-tri-hydroxychalcone	yes	yes		[86]
Grape seed	proanthocyanidin	yes	yes (B16, GP + UV)	antioxidant activity, 8OHdG	[56]
*Aspergillus fumigatus* and *Saccharomyces cerevisiae*	melanin degrading enzymes	-	-		[70]
*Carthamus tinctorius* safflower seeds	1) *N*-feruloylserotonin,2) *N*-(*p*-coumaroyl)serotonin, and 3) acacetin	yes, 1,2 > arbutin	yes (SB, B16). 1,2 > arbutin		[49]
*Glycyrrhiza uralensis*	Glycyrrhisoflavone (1) and glyasperin C(2)	yes	yes (B16) 1 > 2		[87]
*Punica granatum* Pomegranate	ellagic acid	yes ≈ Arb	yes (GP + UV) ≈ AA		[57]
Fish, Poultry	vitamin B3 derivative, niacinamide	no	no	MT inh. Mc/Kc cocult. CT	[53]
*Piper longum*	piperlonguminine	no	yes (B16 + msh)	Tyr mRNA red. cAMP pathway via MITF inh.	[88,89]
*Angelica dahurica*	isoimperatorin imperatorin	no	yes (B16)	Tyr protein + mRNA red.	[90]
*Artocarpus lakoocha* heartwood	oxyresveratrol	yes	Nd	CT (female volunteers) > KA > licorice	[60]
*Astragalus taschkendicus*	askendoside B	yes	Nd		[91]
*Spatholobus suberectus* Dunn (Leguminosae) Chinese herb	Butin (most effective compound)	yes	yes (nHEM)	Tyr,Trp-1 and Trp-2 reduced (WB,qPCR)	[64]
*Sophora japonica* and *Spatholobus suberectus* out of 25 Chinese Herbs	high phenolic content, e.g., gallic acid	yes	yes (nHEM)	AO activity (DPPH)	[92]
*Galla Chinensis Radix Clematidis* out of 90 Chinese Herbs	unknown	yes	yes (Mel-ab, melan-a, melan-a/SP1 cocult.)	Effects on Tyr, Trp-1 and Trp-2expression	[52]
*Kaempferia pandurata*	chalcone compounds, isopanduratin A and 4-hydroxypanduratin A	yes > PTU	yes (melan-a) > PTU	Tyr protein reduced	[93]
Corn bran	Polyamine conjugates, *N,N′*-dicoumaroylputrescine (DCP), *N*-*p*-coumaroyl-*N′*-feruloylputrescine (CFP), and *N,N′*-diferuloyl-putrescine (DFP)	yes DCP > AA	yes (B16) DFP > Arb	AO activity (DPPH)	[94]
*Podocarpus macrophyllus*	2,3-dihydro-4′,4‴-di-*O*-methylamentoflavone	yes	yes (nHEM)	Trp-2 mRNA reduced	[95]
Longan seed	corilagin, gallic acid and ellagic acid or other phenolic/flavonoid glycosides and ellagitannins	yes	n.d.	AO activity (DPPH and ORAC assays)	[71]
*Gastrodia elata Blume Orchidaceae*	(synthetic) *p*-hydroxybenzyl alcohol	yes (Irrev)	yes (B16, mouse MC-KC cocult.)	AO; radical scavenger	[51,96]
*Sophora flavescens*	1) kurarinol, 2) kuraridinol, and 3) trifolirhizin	yes 1,2 > KA 1,2 (nc)	yes (B16)		[97]
*Cucumis sativus*	Lutein	no	yes (B16)	Tyr protein reduced	[98]
*Lespedeza cyrtobotrya*	Haginin A	yes (nc)	yes (melan-a) GP (+UV) zebra fish	MITF, Tyr, Trp-1 reduced. Erk induced	[59]
*Malpighia emarginata* Acerola fruit	cyanidin-3-alpha-*O*-rhamnoside. pelargonidin-3-α-*O*-rhamnoside	yes	yes (B16) GP (+UV)		[11]
Coccoloba uvifera Sea grape	unknown	yes (nHEM)		AO; reduces IL-1alpha, TNF-alpha and alpha-MSH in nHEM + UV	[74]
*Salicornia herbacea*, halophyte		yes	yes (B16)	AO activity	[99]
*Allium* species such as garlic and onions.	1-propylmercaptan	yes ≈ KA	yes ≈ KA		[100]
*Willughbeia coriacea* *Phyllanthus urinaria* out of 14 medicinal plants Central Kalimantan	unknown	yes yes	yes (B16) yes (B16)	AO assay DPPH	[101]
*Rhus Ghinensis*; Chinese galls	3 Gallotannins; 2,3,4,6-tetra-*O*-galloyl-d-glucopyranose, 1,2,3,6-tetra-*O*-galloyl-β-d-glucopyranose, and 1,2,3,4,6-penta-*O*-galloyl-β-d-glucopyranose	yes (nc)	Yes (B16 + UVA; MSH)		[102]
*Rhus succedanea*	10′(Z)-heptadecenyl-hydroquinone	yes > HQ	yes > HQ (B16)		[103]
*Polygonum cuspidatum.* *Paris polyphylla* *Vitex negundo*	Physcion (anthraquinone + anthraquinone analog) (+)-Lyoniresinol	yes ≈ KA yes > KA yes > KA	n.d. n.d. n.d,	Good skin permeation	[10,104] [105]
Raspberry	Tiliroside	yes	Yes (B16)	>Arb	[106]
*Erigeron breviscapus* Chinese herb	(2Z,8Z)-matricaria acid methyl ester	no	yes (B16, elan-a > Arb	Tyr protein reduced?	[107]
*Alpinia galanga* and *Curcuma aromatica* medicinal plants	eugenol and curcuminoids possible active ingredients	yes	yes (G361 ma cells + UVA)	AO defence	[108]
Grape seed	oligomeric proanthocyanidins	-	yes, nHEM + UV	effects on TE, Trp-1 and Trp-2 expression AO activity	[65]

* Modes of action tested; TI; tyrosinase inhibition, (c)competitive (u) uncompetitive (nc) non-competitive and (m) mixed mode; PI; pigment inhibition, SB; Streptomyces bikiniensis, B16 or other melanoma cultures, melan-a mouse melanocytes, nHEM; normal human epidermal melanocytes, SEM; skin equivalent model, (α)-msh; (α)-melanocyte stimulating hormone, UV; ultraviolet, GP; guinea pig + msh or uv induced pigmentation; CT; tested in clinical trial.

** Comparison of effects on tyrosinase inhibition (TI) and pigmentation inhibition (PI) are mostly done in comparison to Arbutin (Arb), Kojic acid (KA) Ascorbic Acid (AA) and phenylthiourea (PTU). Other modes of action; AO; antioxidant; TE; tyrosinase expression (mRNA), MT; melanosome transport; 8OHdg = 8 hydroxy deoxy guanosine.

**Table 3.** Limited selection of whitening products available on the market with some information on active ingredients.

**Company**	**Product**	**Ingredients**	**Documentation; *in Vitro/in Vivo* Effect**
Revitol	Skin Brightener	Arbutin, Lumiskin (diacetyl boldine), Z Whitener (new natural ingredient, unknown) + vitamins A,C and E and other natural extracts (antioxidants)	Lumiskin TM: action on tyrosinase expression based on principle described by Fuller 2000 [128] http://naturalskincareformula.com/
Premium Naturals	Skinbright	Arbutin, Kojic Acid, Lemon Extract	www.whiterskin.com/
Sisquoc	Lucederm	Niacinamide, α-Arbutin, Kojic Acid, Mulberry, Bearberry, Licorice, Lemon	www.whiterskin.com/ [29,53,82,83]
LIBiol	Synerlight	*Actinidia Chinensis* (Kiwi) Fruit, *Sophora Angustifolia* Root	http://www.gattefossecanada.ca/
Bayer HealthCare	Mandresy extract	*Buddleja axillaris* leaves; extract rich in orthocinnamic compounds and flavonoids, verbascoside & luteolin	TI (mushroom); PI (nHEM + UV); reduces dendricity; *in vivo* brightening 8 volunteers (Chromameter) www.serdex-plantextracts.com: United States Patent Application 20090028969
Civant Skin care	Meladerm	Kojic Acid, α-Arbutin, Niacinamide, Mulberry, Bearberry, Licorice, Tego® Cosmo C250, Gigawhite, Lemon Juice, Emblica TegoCosmo; a natural amino acid derivative that belongs to the class of guanidine compounds Giga white:plant extracts from the Swiss alps; *Malva Sylvestris, Mentha Piperita, Primula Veris, Alchemilla Vulgaris, Veronica Officinalis, Melissa Officinalis, Achillea Millefolium*	niacinamide, mulberry and licorice (refs. [29,53,82,83]) www.whiterskin.com/;http://www.whiterskin.com/studies/cosmo.pdfhttp://www.whiterskin.com/studies/giga.pdf
Juju Cosmetics	Tosekki whitening cream	Glycyrrhetinic acid, Ginseng, *Houttuynia*, Yeast, Coix, Horse Chestnut, Arica, Grape Leaf, *Ypericum*, Ivy, Witch Hazel, Sophora Root, Mulberry Bark, Peony Root, Japanese Angelica Root, Rose Fruit and other ingredients.	Glycyrrhetinc acid; Sophora Root; Peony Root; Mulberry Bark (refs. [25,29,38,46,82,83,86]) http://beautyknot.wordpress.com/2009/02/27/juju-cosmetics-tosekki-whitening-cream/
Lipotec	Chromabright	dimetylmethoxy chromanyl palmitate	TI (mushroom + human); PI (nHEM) photoprotective; *in vivo* brightening 20 Asian females (Chromameter) www.lipotec.com ; Patent; http://www.maxworth.co.th/max/pdf/ES291%20Chromabriht.pdf

## Abbreviations

AA: Ascorbic Acid
ACTH: adrenocorticotropic hormone
AO: antioxidant
Arb: Arbutin
bFGF: basic fibroblast growth factor
BMP: bone morphogenetic proteins
cAMP: cyclic AMP
CT: clinical trials
DNJ: 1-deoxynojirimycin
DPPH: 1,1-diphenyl-2-picrylhydrazyl
ET-1: endothelin-1
ERK: extracellular signal-regulated kinases
GP: guinea pig
HGF: hepatocyte growth factor
HQ: hydroquinone
IC50: half maximal inhibitory concentration
Il1α: interleukin 1α
KA: kojic acid
l-DOPA: l-dihydroxyphenylalanine
MC1R: melanocortin-1 receptor
MITF: microphtalmia transcription factor
(α)-msh: (α)-melanocyte stimulating hormone
MT: melanosome transport
nHEM: normal human epidermal melanocytes
8OHdg: 8 hydroxy deoxy guanosine
ORAC: oxygen radical absorbance capacity
PKA: protein kinase A
PKC: protein kinase C
PPAR: peroxisome proliferator- activated receptor
PTU: phenylthiourea
SAR: structure activity relationship
sAPP: soluble N-terminal ectodomain of the beta-amyloid precursor protein
SCF: stem cell factor
SEM: skin equivalent model
Sox: Sry-related HMG box
TE: tyrosinase expression
TI: tyrosinase inhibition
(c): competitive mode
(nc): non competitive mode
(m): mixed mode of inhibition
TNF-α: tumor necrosis factor-α
TRP: tyrosinase related protein
UV: ultraviolet
UVA: ultraviolet A
UVB: ultraviolet B
UVR: ultraviolet radiation
